# Osimertinib, a third‐generation EGFR tyrosine kinase inhibitor: A retrospective multicenter study of its real‐world efficacy and safety in advanced/recurrent non‐small cell lung carcinoma

**DOI:** 10.1111/1759-7714.13378

**Published:** 2020-03-04

**Authors:** Takayuki Kishikawa, Takashi Kasai, Masahiko Okada, Ichiro Nakachi, Sayo Soda, Ryo Arai, Ayako Takigami, Masafumi Sata

**Affiliations:** ^1^ Department of Respiratory Medicine Tochigi Cancer Center Utsunomiya Japan; ^2^ Pulmonary Division, Department Internal Medicine Saiseikai Utsunomiya Hospital Utsunomiya Japan; ^3^ Department of Pulmonary Medicine and Clinical Immunology Dokkyo Medical University School of Medicine Shimotuga‐Gun Japan; ^4^ Division of Pulmonary Medicine, Department of Medicine Jichi Medical University Hospital Shimotsuke‐City Japan

**Keywords:** Epidermal growth factor receptor T790M mutation, non‐small cell lung cancer, osimertinib, tyrosine kinase inhibitor

## Abstract

**Background:**

Osimertinib is recommended for T790M mutation‐positive advanced non‐small cell lung cancer (NSCLC) resistant to first‐ and second‐generation epidermal growth factor receptor (EGFR)‐tyrosine kinase inhibitors (TKIs). Recently, some reports exist on the real‐world use of osimertinib; however, reports involving third/later‐line use are few. Hence, this study was conducted to evaluate the efficacy and safety of osimertinib used in various treatment lines for T790M‐positive NSCLC patients.

**Methods:**

This retrospective, observational, multicenter study included T790M‐positive advanced/recurrent NSCLC patients treated with osimertinib from May 2016 to March 2018. The clinical characteristics, efficacy, and adverse events were retrospectively investigated. The Kaplan‐Meier method was used to analyze progression‐free survival (PFS) and overall survival (OS). PFS‐associated clinical characteristics were evaluated using the Cox proportional hazards model.

**Results:**

The objective response rate (ORR) and disease control rate (DCR) were 60.7% and 91.1%, respectively; the median PFS was 11.0 months. There were no significant differences in the median PFS for patients treated with osimertinib as second‐line and third−/later‐line (14.5 vs. 11.0 months respectively, *P* = 0.327). Analysis using the Cox proportional hazards model for clinical features affecting PFS also revealed no significant factors. Adverse events of grade ≥ 3 were reported in 15 patients (26.8%); the most common were anemia (*n* = 3) and cutaneous toxicity (*n* = 3). Grade 4 neutropenia was observed in one patient; any‐grade pneumonitis was observed in six patients (10.7%), including one with grade 3 pneumonitis.

**Conclusions:**

Osimertinib demonstrated efficacy even when administered as third−/later‐line treatment to NSCLC patients. Osimertinib‐related pneumonitis was observed more frequently than previously reported.

**Key points:**

**Significant findings of the study**

Osimertinib shows efficacy even as later‐line treatment in T790M mutation‐positive NSCLC patients previously treated with EGFR‐TKIs. However, the incidence of ≥ grade 3 adverse events, especially pneumonitis, was higher than that previously reported by other studies.

**What this study adds**

Osimertinib was approved for previously EGFR‐TKI‐treated EGFR T790M‐positive NSCLC. With the increasing frequency of its use as first‐line treatment, this study provides valuable evidence for the efficacy and safety of osimertinib for previously EGFR‐TKI‐treated NSCLC.

## Introduction

The 2018 National Comprehensive Cancer Network (NCCN) Clinical Practice Guidelines for Non‐Small Cell Lung Cancer (NSCLC) recommend epidermal growth factor receptor (EGFR)‐tyrosine kinase inhibitors (TKIs) as first‐line treatment for unresectable, *EGFR* mutation‐positive, advanced NSCLC.^1^ It is known that lung cancer patients with activated *EGFR* mutations respond to first‐ or second‐generation EGFR‐TKIs, such as gefitinib, erlotinib, and afatinib.^2^, ^3^, ^4^, ^5^, ^6^, ^7^ However, in many cases, the effects are transient, cause drug resistance, and lead to deterioration in clinical conditions in approximately one year.^8^ Approximately 50% of drug resistance events are caused by the emergence of drug resistance mutations, such as the T790M mutation, against a background of an activated *EGFR* mutant.^9^, ^10^ The T790M mutation substitutes a threonine with a methionine at position 790 of exon 20, thus affecting the ATP‐binding site of the receptor tyrosine kinase. Osimertinib is a third‐generation EGFR‐TKI that targets the T790M mutation. In a phase III trial (AURA3), osimertinib was linked to a significantly longer progression‐free survival (PFS), compared to standard chemotherapy with platinum and pemetrexed, as second‐line treatment for T790M mutation‐positive advanced NSCLC (median PFS 10.1 vs. 4.4 months).^11^ Therefore, in the case of treatment resistance to first‐ or second‐generation EGFR‐TKIs, after testing for the *EGFR* T790M mutation, osimertinib treatment is recommended as second‐line treatment for T790 mutation‐positive patients. More recently, in a phase III trial (FLAURA),^12^ osimertinib showed a longer PFS than gefitinib or erlotinib when administered as first‐line treatment; moreover, osimertinib has been used as first‐line treatment in the presence/absence of the T790M mutation in clinical practice. However, the efficacy and safety of osimertinib in clinical practice, used often as later‐line treatment, remain unclear. Therefore, we retrospectively evaluated patient characteristics, treatment status, and the efficacy and safety of osimertinib treatment in clinical practice through a multicenter evaluation of patients who received osimertinib for previously EGFR‐TKI‐treated advanced/recurrent T790M‐positive NSCLC.

## Methods

### Patient eligibility and data collection

A total of 56 patients with EGFR T790M‐positive advanced or recurrent NSCLC (stage IIIB or IV), who had received osimertinib as second−/later‐line treatment in one of four hospitals belonging to the Tochigi‐kitakan Thoracic Oncology Research Organization (TOTORO) from 1 May 2016 to 31 March 2018, were included in this study. The following characteristics of the 56 patients were collected from medical reports obtained from the hospitals: demographic information, smoking status, renal function, Eastern Cooperative Oncology Group performance status (ECOG‐PS), tumor histology, clinical stage, the presence/absence of central nervous system (CNS) metastasis, *EGFR* mutations at diagnosis, testing method to confirm T790M mutations before osimertinib treatment, number of previous treatments before osimertinib, initial dose of osimertinib, subsequent dose reduction and discontinuation, the duration of administration, efficacy data of osimertinib, and adverse events.

### Efficacy and safety evaluation

Objective response rate (ORR) and disease control rate (DCR) were assessed according to the Response Evaluation Criteria in Solid Tumors (RECIST) version 1.1 to examine the clinical efficacy of osimertinib.^13^ The Kaplan‐Meier method and log‐rank test were used to analyze PFS and overall survival (OS). The median PFS values were compared between the groups of patients classified according to treatment line (second‐line vs. third−/later‐line and second‐line vs. fourth−/later‐line), to assess the impact of osimertinib treatment sequence on PFS. In addition, clinical characteristics associated with the PFS with osimertinib were evaluated using the Cox proportional hazards model.

Osimertinib‐related adverse events were also evaluated according to the Common Terminology Criteria for Adverse Events version 4.0 (CTCAE v4.0).^14^ A logistic regression analysis was performed to assess the factors associated with severe adverse events.

This retrospective study was approved by the Institutional Review Board of the Tochigi Cancer Center (No. A‐458). Owing to the retrospective nature of the study, the requirement for informed consent was waived according to the Japanese ethical guidelines for clinical research.

All statistical analyses were performed with EZR (Saitama Medical Center, Jichi Medical University, Saitama, Japan), which is a graphical user interface for R (The R Foundation for Statistical Computing, Vienna, Austria); moreover, it is a modified version of R Commander designed to add statistical functions that are frequently used in biostatistics.^15^

## Results

### Patient characteristics

In total, 56 patients who received osimertinib treatment were registered at the four hospitals. Patient demographics are listed in Table 1. The median age at the time of osimertinib treatment was 69.5 years (range, 39–91 years); 17 patients were male; 39 were female; and 34 patients (60.7%) had a history of smoking. The ECOG‐PS in this study was as follows: 0 (28.6%), one (55.3%), two (12.5%), and three (3.6%). Histologically, except for one patient with adenocarcinoma and large cell carcinoma, all the other patients had adenocarcinoma at initial diagnosis. The clinical stage before osimertinib treatment was as follows: IIIB (7.1%), IV (73.2%), and postoperative recurrence (19.6%); 20 patients (35.7%) had CNS metastases. *EGFR* mutations at initial diagnosis were as follows: exon 19 deletion‐positive (*n* = 28, 50.0%) and L858R point mutation‐positive (*n* = 28, 50.0%). Two of the L858R point mutation‐positive patients were de novo T790M mutation‐positive. The following methods were used to detect the T790M mutation prior to osimertinib treatment: rebiopsy specimens from diseased tissue (*n* = 36, 64.3%), cytological specimens such as pleural effusion (*n* = 11, 19.6%), plasma specimens (*n* = 5, 8.9%), and both tissue and plasma specimens (*n* = 2).

**Table 1:** Patient characteristics

	Patients (*n* = 56)
Characteristics	*n* (%)
Age (years), median (range)	69.5 (39–91)
< 75 years	35 (62.5)
≥ 75 years	21 (37.5)
Gender	
Male	17 (30.4)
Female	39 (69.6)
Smoking history	
Never	34 (60.7)
Former/current	21 (37.5)
Unknown	1 (1.8)
BSA (m^2^)	
≥1.5	27 (48.2)
<1.5	29 (51.8)
CCr (mL/min)† or eGFR (mL/min/1.73 m^2^)‡	
≥60	29 (51.8)
<60	27 (48.2)
ECOG PS	
0	16 (28.6)
1	31 (55.3)
2	7 (12.5)
3	2 (3.6)
4	0 (0.0)
Stage	
IIIB	4 (7.1)
IV	41 (73.2)
Postoperative recurrence	11 (19.6)
CNS metastasis	20 (35.7)
Histological type at initial diagnosis	
Adenocarcinoma	55 (98.2)
Adenocarcinoma and large‐cell carcinoma	1 (1.8)
*EGFR* mutation types at initial diagnosis	
Exon 19 deletion	28 (50.0)
L858R	28 (50.0)
T790M (de novo)§	2 (3.6)
Samples with T790M	
Tissue	36 (64.3)
Cytology	11 (19.6)
Plasma	5 (8.9)
Both tissue and plasma	2 (3.6)

† CCr was calculated using the Cockcroft and Gault (CG) equation for glomerular filtration rate (GFR) estimation.

‡ eGFR was determined using the Chronic Kidney Disease Epidemiology Collaboration (CKD‐EPI) creatinine‐based equation.

§ Two patients were identified as having double *EGFR* mutations (L858R and T790M).

BSA, body surface area; CCr, creatinine clearance; CNS, central nervous system; ECOG, Eastern Cooperative Oncology Group; EGFR, epidermal growth factor receptor; eGFR, estimated glomerular filtration rate; PS, performance status.

The treatments administered before and after osimertinib are shown in Table 2. All patients were treated with first‐ or second‐generation EGFR‐TKIs: gefitinib (*n* = 38, 67.9%), erlotinib (*n* = 28, 50.0%), and afatinib (n = 21, 37.5%). A total of 23 patients (41.1%) were treated with two or more EGFR‐TKIs, and three patients (5.4%) were rechallenged with the same type of TKI. Moreover, 35 patients (62.5%) received treatment with cytotoxic agents. A total of 33 patients (58.9%) received platinum combination therapy, and one patient (1.8%) received antiprogrammed cell death protein‐1 (PD‐1) antibody. The osimertinib treatment line was as follows: second‐line (*n* = 19, 33.9%), third‐line (*n* = 15, 26.8%), and fourth−/later‐line (*n* = 22, 39.3%). The median number of previous regimens was two (range, 1–14). Osimertinib (80 mg/day) was orally administered to all patients.

**Table 2:** Treatments received

	Patients (*n* = 56)
Received before osimertinib	*n* (%)
EGFR‐TKIs	56 (100)
Gefitinib	38 (67.9)
Erlotinib	28 (50.0)
Afatinib	21 (37.5)
EGFR‐TKI rechallenge	3 (5.4)
Cytotoxic agents	35 (62.5)
Platinum combination therapy	33 (58.9)
Anti‐PD‐1 antibody	1 (1.8)
Number of previous regimens (median, range)	2 (1–14)
1	19 (33.9)
2	15 (26.8)
≥ 3	22 (39.3)
Received after osimertinib	Patients (n = 26)
	Number (%)
Cytotoxic agents	16 (61.5)
EGFR‐TKIs	11 (42.3)
Gefitinib	4 (15.4)
Erlotinib	6 (23.1)
Afatinib	4 (15.4)
Osimertinib	1 (3.8)
Anti‐PD‐1 antibody	6 (23.1)

EGFR, epidermal growth factor receptor; PD‐1, programmed death protein‐1; TKI, tyrosine kinase inhibitor.

### Clinical outcomes

**Figure 1:**
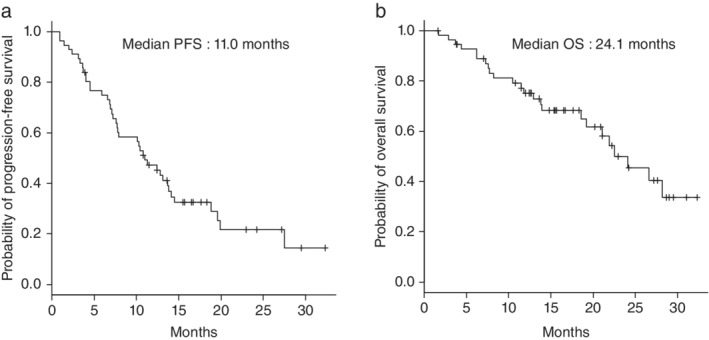
Kaplan‐Meier curves for (**a**) progression‐free survival (PFS) and (**b**) overall survival (OS) in the study population (median duration of follow‐up: 15.1 months).

**Figure 2:**
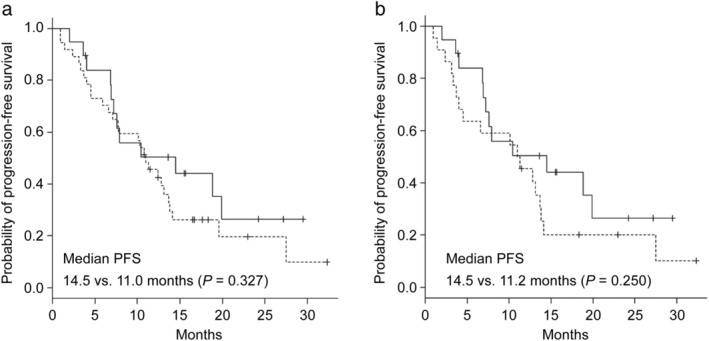
Kaplan‐Meier curves for progression‐free survival (PFS) in the patients who received (**a**) osimertinib as second‐line or third‐/later‐line (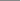) second‐line (*n* = 19), and (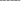) third‐/later‐line (*n* = 37) and (**b**) osimertinib as second‐line or fourth‐/later‐line (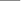) second‐line (*n* = 19), and (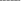) fourth‐/later‐line (*n* = 22).

The median follow‐up duration from the start of osimertinib treatment was 15.1 months (range, 1.6–32.3 months). At the time of final analysis, 17 patients (30.4%) were still receiving osimertinib. A total of 24 patients (42.9%) died during the follow‐up period; of these, except for one death caused by another disease, all other patients died of the primary disease. The median duration of osimertinib treatment was 10.8 months (range, 0.7–32.3 months). The best outcomes determined according to the RECIST guideline were as follows: complete response (CR, *n* = 2, 3.6%), partial response (PR, *n* = 32, 57.1%), stable disease (SD, *n* = 17, 30.4%), progressive disease (PD, *n* = 4, 7.1%), and not evaluable (NE, *n* = 1, 1.8%). ORR was 60.7% (95% confidence interval [CI] 46.8–73.59), and DCR was 91.1% (95% CI 80.4–97.0). The median PFS was 11.0 months (95% CI 7.6–13.8), and the median OS was 24.1 months (95% CI 18.6‐not calculable) (Table 3). Figure 1 shows the Kaplan‐Meier curves for the PFS (Fig 1a) and OS (Fig 1b) of the study population. The median PFS was not significantly different (14.5 vs. 11.0 months, *P* = 0.327) between the groups of patients classified by treatment line (second‐line [*n* = 19, 33.9%] vs. third−/later‐line [*n* = 15, 26.8%]) (Fig 2a). Similar results were obtained for the median PFS of the other groups (second‐line vs. fourth−/later‐line [n = 22, 39.3%]) (14.5 vs. 11.2 months, *P* = 0.250) (Fig 2b). In addition, the prognostic value of the clinical characteristics was assessed using the Cox proportional hazards model for univariate analyses of PFS. The patient population with PS ≥ 2 tended to show poor PFS; however, the difference was not statistically significant (Table 4).

**Table 3:** Clinical efficacy data of osimertinib treatment

Treatment response	All patients (*n* = 56) (%)	95% CI
CR	2 (3.6)	
PR	32 (57.1)	
SD	17 (30.4)	
PD	4 (7.1)	
NE	1 (1.8)	
ORR (%)	34 (60.7)	46.8–73.5
DCR (%)	51 (91.1)	80.4–97.0

CI, confidence interval; CR, complete response; DCR, disease control rate; NE, not evaluated; ORR, objective response rate; PD, progressive disease; PR, partial response; SD, stable disease.

**Table 4:** Univariate analysis of associations between PFS and clinical features

	Univariate hazard ratio (95% CI)	*P*‐value
Age (< 75 vs. ≥ 75)	0.689 (0.344–1.381)	0.294
Smoking history (No vs. Yes)	1.102 (0.578–2.104)	0.767
BSA (m^2^) (≥ 1.5 vs. < 1.5)	1.314 (0.702–2.459)	0.394
CCr (mL/min)† or eGFR (mL/min/1.73 m^2^)‡ (≥ 60 vs. < 60)	1.301 (0.697–2.428)	0.408
ECOG PS (0–1 vs. ≥ 2)	2.020 (0.876–4.657)	0.099
CNS metastasis (No vs. Yes)	1.065 (0.561–2.021)	0.848
EGFR mutation types (exon19 del vs. L858R)	1.080 (0.580–2.011)	0.808
Treatment lines (second‐ vs. third−/later‐line)	1.403 (0.710–2.775)	0.330

† CCr was calculated using the Cockcroft and Gault (CG) equation for glomerular filtration rate (GFR) estimation.

‡ eGFR was determined using the Chronic Kidney Disease Epidemiology Collaboration (CKD‐EPI) creatinine‐based equation.

BSA, body surface area; CCr, creatinine clearance; CNS, central nervous system; ECOG, Eastern Cooperative Oncology Group; EGFR, epidermal growth factor receptor; eGFR, estimated glomerular filtration rate; PFS, progression‐free survival; PS, performance status.

A total of 26 patients (46.4%) received some treatment after osimertinib (Table 2). Most of them received cytotoxic agents; 11 patients underwent rechallenge with first‐ or second‐generation EGFR‐TKIs. In addition, one patient received a rechallenge with osimertinib. Six patients received anti‐PD‐1 antibodies. In this study, no detailed investigation was conducted regarding the efficacy of treatment after osimertinib.

### Adverse events

A total of 50 patients (89.3%) reported at least one adverse event, and 15 patients (26.8%) reported all‐causality ≥ grade 3 adverse events. Grade 4 neutropenia was observed in one patient (Table 5). The major ≥ grade 3 adverse events were anemia (*n* = 3), cutaneous toxicity (*n* = 3), stomatitis (*n* = 2), leukopenia (*n* = 2), neutropenia (*n* = 2), and aspartate aminotransferase/alanine aminotransferase elevation (*n* = 2). A total of 20 patients (35.7%) underwent dose reduction or a transient washout due to adverse events, and 10 patients (17.9%) discontinued treatment due to adverse events. Any‐grade pneumonitis was observed in six patients (10.7%), including one with grade 3 pneumonitis, and osimertinib treatment was discontinued in all patients. There was no treatment‐related death. Multivariate logistic regression analysis was performed to assess the factors associated with severe adverse events. The incidence tended to be high in patients aged ≥ 75 years; however, no statistically significant differences were observed (Table 6).

**Table 5:** Osimertinib‐related adverse events

	Any grade	≥ Grade 3
Events	Patients, *n* (%)	
All events	50 (89.3)	15 (26.8)
Hematologic toxicity		
Anemia	21 (37.5)	3 (5.4)
Thrombocytopenia	14 (25.0)	0
Leukopenia	12 (21.4)	2 (3.6)
Neutropenia	4 (7.1)	2 (3.6)†
Hypoalbuminemia	15 (26.8)	1 (1.8)
AST/ALT elevation	14 (25.0)	2 (3.6)
Creatinine elevation	12 (21.4)	0
Hyperbilirubinemia	1 (1.8)	0
Nonhematologic toxicity		
Cutaneous toxicity (rash, paronychia, and dry skin)	20 (35.7)	3 (5.4)
Fatigue	7 (12.5)	0
Diarrhea	7 (12.5)	0
Pneumonitis	6 (10.7)	1 (1.8)
Stomatitis	6 (10.7)	2 (3.6)
Anorexia	4 (7.1)	0
Nausea	2 (3.6)	0
Alopecia	2 (3.6)	0
Fever	1 (1.8)	0
Myalgia	1 (1.8)	0
Lung infection	1 (1.8)	0
Hypertension	1 (1.8)	0
Ventricular arrhythmia	1 (1.8)	0
QTc prolongation	0	1 (1.8)
Depression	0	1 (1.8)

† Grade 4 neutropenia was observed in one patient. Adverse events were graded according to the National Cancer Institute Common Terminology Criteria for Adverse Events version 4.0.

**Table 6:** Multivariate logistic regression analysis of factors associated with ≥ grade 3 adverse events

	Odds ratio (95% CI)	*P*‐value
Age (< 75 vs. ≥ 75)	3.440 (0.804–14.700)	0.096
Smoking history (No vs. Yes)	1.520 (0.317–7.290)	0.601
BSA (≥ 1.5 vs. < 1.5)	2.040 (0.427–9.710)	0.372
CCr (mL/min)† or eGFR (mL/min/1.73 m^2^)‡ (≥60 vs. <60)	0.487 (0.113–2.110)	0.336
ECOG PS (0–1 vs. ≥ 2)	2.230 (0.386–12.900)	0.370
Treatment lines (second‐ vs. third−/later‐line)	1.340 (0.313–5.770)	0.691

† CCr was calculated using the Cockcroft and Gault (CG) equation for glomerular filtration rate (GFR) estimation.

‡ eGFR was determined using the Chronic Kidney Disease Epidemiology Collaboration (CKD‐EPI) creatinine‐based equation.

BSA, body surface area; CCr, creatinine clearance; ECOG, Eastern Cooperative Oncology Group; eGFR, estimated glomerular filtration rate; PFS, progression‐free survival; PS, performance status.

## Discussion

EGFR‐TKIs are the standard treatment for *EGFR* mutation‐positive advanced NSCLC.^2^, ^3^, ^4^, ^5^, ^6^, ^7^ The ORR and median PFS in patients treated with a first‐ or second‐generation EGFR‐TKI as first‐line treatment have been reported to be 56%–74% and 9.2–13.1 months, respectively.^2^, ^3^, ^4^, ^5^, ^6^, ^7^, ^16^, ^17^ Although these patients showed excellent responses to first‐ and second‐generation EGFR‐TKIs and improved prognosis, almost all patients had become resistant to treatment over time. T790M mutations have been reported to be involved in about half of the cases of resistance.^9^, ^10^ A previous study had shown the efficacy of osimertinib as second‐line treatment for T790M mutation‐positive NSCLC.^11^ Osimertinib was approved in 2016 for manufacture and sale in Japan for previously EGFR‐TKI‐treated EGFR T790M‐positive NSCLC and has been used in various treatment lines, not limited to the second line. Furthermore, osimertinib was approved for expanded indication as first‐line treatment in 2018; it is expected that the frequency of its use as later‐line treatment will decrease in the future. Therefore, this study provides valuable evidence for the efficacy and safety of osimertinib for previously EGFR‐TKI‐treated NSCLC in the absence of such treatment data.

In this retrospective study, ORR and median PFS were 60.7% and 11.0 months, respectively. This study included nine patients with PS 2/3 and many elderly patients; moreover, although osimertinib was administered as second‐line treatment to only 19 patients (33.9%), its efficacy was similar to that observed in the AURA3 trial (ORR 71% and median PFS 10.1 months).^11^ There were no significant differences in median PFS between the second‐line group (*n* = 19) and the third−/later‐line group (*n* = 37), including 22 patients (39.3%) who received osimertinib as fourth−/later‐line treatment. Additionally, in this study, two cases of complete response (CR) were observed: one patient received osimertinib as second‐line treatment and the other as third‐line. These results suggest that osimertinib shows clinical efficacy even when administered to patients as a subsequent treatment.

A total of 15 patients (26.8%) had ≥ grade 3 adverse events, most of which were hematologic toxicity (eg, anemia, leukopenia, and neutropenia). Although the adverse event profile was similar to that seen in previous studies, the incidence was more frequent.^11^, ^12^, ^18^, ^19^ We examined the incidence of ≥ grade 3 adverse events and its association with patient characteristics, such as the age, body surface area, renal function, and the number of regimens before osimertinib treatment. The incidence tended to be higher in patients aged ≥ 75 years than in patients aged < 75 years (*P* = 0.096); no association with other significant factors was observed. Moreover, in this population, six patients (10.7%) had any‐grade pneumonitis, including one patient with grade 3 pneumonitis, which was a higher frequency than that observed in previous studies.^11^, ^12^, ^18^, ^19^ The administration of osimertinib was discontinued in these patients; no case had any lethal outcome. It is widely recognized that the incidence of drug‐induced pneumonia is greater in Japan than in other regions, but the mechanism is unknown.^20^ Previous first‐generation EGFR‐TKI studies suggest that pulmonary injury in Japanese patients is associated with smoking history, poor PS, preexisting pulmonary fibrosis, and prior treatment with chemotherapy.^21^, ^22^ Moreover, recent reports have described an increased incidence of interstitial lung disease in patients treated with osimertinib after treatment with anti‐PD‐1 antibody.^23^, ^24^, ^25^ In this study, neither did any patient have interstitial lung disease as the underlying disease nor did the one patient who received anti‐PD‐1 antibody prior to osimertinib have pneumonitis. Although statistical analysis was not performed on the factors that caused the incidence of pneumonitis due to the small number of samples, the pneumonitis may have been due to osimertinib being administered as relatively late‐line treatment. Further investigation into a higher number of cases would be necessary to examine this phenomenon.

Recently, several similar retrospective studies of osimertinib in a real world data for treated NSCLC have been reported.^26^, ^27^ The efficacy data in this study highlight the relevance of patient characteristics to real‐world clinical practice. Although many patients were treated with osimertinib as third−/later‐line treatment, the ORR and PFS were almost similar to those observed in the randomized controlled trials. Similar to our study, in a recent retrospective study involving a large number of patients receiving osimertinib as a third−/late‐line, median PFS were 8.5 months (95% CI, 7.4 to 9.6) in second‐line group, 9.1 months (95% CI, 6.6 to 11.6) in third/late‐line group, respectively. In that study, only 77 out of 94 cases were confirmed to be positive for the T790M mutation, so a simple comparison was impossible due to differences in population, but our efficacy data was better.

However, toxicity such as pneumonitis was observed more frequently, necessitating the earlier discontinuation of osimertinib treatment.

There are few reports of clinical data on such late‐line osimertinib treatment, making the design of prospective studies considering treatment sequence and long‐term prognosis difficult. Therefore, the investigation of the efficacy and safety of osimertinib as a late‐line treatment for previously treated EGFR‐TKI‐treated NSCLC patients depends on retrospective data. To the best of our knowledge, our study provides invaluable evidence for the efficacy and safety of osimertinib in the real world. However, the small sample size and short observation period are limitations of this study. Hence, a higher number of cases and long‐term data follow‐up are desirable. In conclusion, our study shows that osimertinib may improve PFS and OS even in a population that includes many patients who received osimertinib as a third−/later‐line treatment. This real‐world evidence may help answer the clinical question of the optimal treatment sequence of osimertinib.

## Disclosure

The authors declare they have no conflict of interest.
